# Foetal scalp blood sampling and ST-analysis of the foetal ECG for intrapartum foetal monitoring: a restricted systematic review

**Published:** 2020-03-27

**Authors:** HMI Demaegd, EGR Bauters, GH Page

**Affiliations:** Jan Yperman Ziekenhuis, Department Gynaecology and Obstetrics, Briekestraat 12, 8900 Ieper.

**Keywords:** foetal scalp blood sampling, ST-analysis, metabolic acidosis, intrapartum monitoring, labour, STAN ®

## Abstract

**Background:**

To investigate if foetal scalp blood sampling (FBS) is useful in preventing foetal metabolic acidosis in labour when ST-analysis of the foetal ECG (STAN ® ) is already being used as a second line technique for intrapartum foetal monitoring with cardiotocography (CTG).

**Design:**

Restricted systematic review.

**Methods:**

Based on a literature search in July 2019, a restricted systematic review was performed. Studies comparing CTG+STAN ® +FBS with CTG+STAN ® , CTG+FBS or CTG only were included. Observational studies allowing FBS in addition to STAN ® reporting the indications, results and neonatal outcomes were included as well.

**Results:**

Five randomised controlled trials (RCT) and seven observational trials were analysed. Based on the analysis of data coming from one RCT, FBS identifies foetal acidosis in 9.9% when performed in specific situations. Similarly, in observational trials it was found that in up to 10% of cases where STAN ® registration was less reliable, FBS suggested foetal acidosis. However, there is no evidence that FBS in these cases was capable of preventing metabolic acidosis or its neurological consequences.

**Conclusion:**

Based on the available literature, no recommendations in favour of combining FBS with STAN ® monitoring can be made.

## Introduction

The aim of foetal monitoring during labour is to identify inadequately oxygenated foetuses in a timely manner and thus avoiding the occurrence of perinatal asphyxia and its serious neurological consequences. For this reason, cardiotocography (CTG) was developed and introduced in 1968. Although CTG is a very sensitive technique (i.e. a reassuring foetal heart rate pattern is a good predictor of foetal wellbeing), its specificity is low (i.e. only a small proportion of foetuses with an abnormal heart rate trace are truly hypoxic) (Ayres-de-Campos et al., 2015).

In 1961, Erich Salingfirst described foetal scalp blood sampling (FBS) to identify foetuses in distress in the presence of abnormal foetal heart rate on intermittent auscultation (Saling, 1981). Its commercialisation in 1997 adjuvant to CTG, was a first attempt to reduce the false-positive rate of CTG by trying to identify those foetuses with true metabolic acidosis and in this way avoiding unnecessary operative interventions. In 2000, ST- analysis of the foetal ECG (STAN ® , Neoventa Medical, Gothenburg, Sweden) was introduced in combination with intrapartum CTG for the same purpose (Visser et al., 2015). Although the results of clinical trials comparing STAN ® monitoring with CTG alone have been conflicting, it is currently being used in most obstetric centres in Flanders (Belgium) as the technique of choice when a more intensive foetal monitoring technique is required.

To date, there are no studies directly comparing FBS and STAN ® monitoring as second line techniques adjuvant to CTG for intrapartum monitoring. Moreover, most studies comparing CTG alone with STAN ® -monitoring adjuvant to CTG allow for FBS “if indicated”. The aim of this restricted systematic review was to investigate if FBS is still useful when STAN ® monitoring is already being used as a second line technique for intrapartum foetal monitoring.

## Background

### Foetal response to hypoxia

The exact pathophysiology of the foetal response to hypoxia remains partially unknown, as most of the current knowledge is based on experiments on sheep. When hypoxaemia persists, an anaerobic cell metabolism is activated in peripheral tissues. As a result, glycogen (stored in the liver and myocardium) is being processed to produce glucose, hereby producing lactate, leading to metabolic acidosis (Garabedian et al., 2017).

The pH-value of foetal blood outside labour is approximately 7.35. During labour, a physiological fall in foetal blood pH occurs. Mean pH on umbilical cord arterial blood postpartum is 7.25. Mild acidosis is defined as an arterial pH lower than 7.15, a pH lower than 7.00 is called severe acidosis (Goldaber et al., 1991; Winkler et al., 1991; Carbonne et al., 2016). Two types of foetal acidosis may occur. Respiratory acidosis is a consequence of the accumulation of CO2 due to an insufficient placental elimination. Typically it is an intermittent event caused by pressure on the umbilical cord during a uterine contraction. Besides a lowered pH, a higher PCO2 (≥75 mmHg) is found on umbilical cord arterial blood postpartum. CO2 is a waste product of foetal metabolism and transported through the placenta to be eliminated via maternal lungs and kidneys. This transplacental transport is interrupted when the umbilical arteries are occluded. Respiratory acidosis dissolves quickly after birth since breathing of the neonate eliminates CO2 and it has no long-term neurological sequelae. Contrary, metabolic acidosis is the consequence of the activation of anaerobic cell metabolism in the presence of hypoxia. Even after the correction of hypoxia, metabolic acidosis can persist for several hours. It is a predictor of severe neonatal morbidity and mortality (Amer- Wåhlin et al., 2019). A pH lower than 7.00 (as this is the cut-off value correlating with an increasing risk of neurological consequences), a base deficit > 12 mmol/L and/or a lactate value > 10 mmol/L on umbilical cord arterial blood are considered markers for severe metabolic acidosis (Garabedian et al., 2017; Carbonneet al., 2016). However, most clinical trials examining the effect of intrapartum foetal monitoring on neonatal outcome use pH <7.05 and a base deficit >12 mmol/L as outcome values. In the literature, the incidence of neonatal metabolic acidosis varies between 1.3% and 3.5% (Westgate et al., 1993; Amer-Wåhlin et al., 2001; Vayssière et al., 2007; Westerhuis et al., 2010)

### Foetal blood sampling (FBS)

In the presence of an abnormal foetal heart rate trace, some guidelines recommend foetal scalp blood sampling as a second-line technique to correctly identify hypoxic foetuses (ACOG, 2009). Initially, scalp pH was used to detect foetal acidosis. A pH of >7.25 is normal, whereas a pH of 7.20- 7.25 is considered suboptimal and a value of <7.20 is abnormal and requires intervention (Garabedian et al., 2017).

Nowadays, a test-strip for estimation of scalp lactate is mostly used, which has several benefits over pH-analysis. It identifies more adequately the metabolic origin of the foetal acidosis, has a significantly lower failure rate and the result is known much faster. The cut-off value for intervention most widely used is a lactate level of 4.8 mmol/L or more. Lactate values between 4.2 and 4.8 mmol/L are considered borderline. A lactate level of 4.2 mmol/L or less is reassuring (https://www.nice.org.uk/guidance/cg190/chapter/recommendations).

Known complications of FBS are laceration, bleeding, hematoma, abscess, meningitis, retention of scalpel fragments and drainage of cerebrospinal fluid upon incision of the fontanelle (Schaap et al., 2011).

Only one randomised controlled trial concerning FBS has been published so far (Haverkamp et al., 1978). In this trial high-risk obstetric patients were assigned to either intermittent auscultation, continuous CTG or CTG combined with FBS for foetal monitoring during labour. No difference in immediate neonatal outcomes (Apgar scores, cord blood gases, neonatal morbidity and mortality) was found between the three groups. The caesarean section rate was significantly increased in the electronically monitored groups. A higher (though not statistically significant) rate of caesarean sections was found in the group monitored with CTG alone (18%), compared to the CTG+FBS group (11%) (Haverkamp et al., 1979). Another randomised controlled trial (the FLAMINGO trial) comparing CTG with CTG+FBS in a mixed obstetric population, with caesarean section rate as primary outcome measure, is ongoing in a tertiary Australian obstetric centre (East et al., 2015).

Thus, most evidence on FBS efficacy consists on observational data. Several studies have demonstrated a high negative predictive value (NPV) of FBS for foetal acidemia at birth (Kruger et al., 1999; Bowler and Beckmann, 2014). However, the correlation between scalp and umbilical cord lactate seems to depend on the interval between scalp sampling and birth (Choserot et al., 2014). FBS has also been shown to correlate with neonatal Apgar scores (Kruger et al., 1999).

One study considered the association of foetal scalp lactate with developmental outcomes during childhood: children with higher lactate levels had an increased probability of fine motoric and cognitive dysfunction at the age of four (Wiberg et al. 2018). Another observational cohort study found that the addition of FBS to continuous CTG in the presence of an abnormal foetal heart rate pattern resulted in an increase in spontaneous births as well as better short-term neonatal outcomes (Stein et al., 2006).

### ST-analysis of the foetal ECG

ST-analysis of the foetal ECG (STAN ® , Neoventa Medical, Gothenburg, Sweden) is another second line technique for intrapartum foetal monitoring. Combining ST-analysis with standard CTG interpretation aims to identify hypoxic foetuses more accurately than CTG alone.

The STAN ® method identifies changes in the ST-interval of the foetal ECG that occur in the presence of foetalcentral hypoxia. An imbalance between myocardial oxygen supply and cardiac work normally leads to activation of anaerobic metabolism. The resulting cardiac glycogenolysis in the foetal myocardium results in hyperkalaemia, which in turn leads to an increase in T-wave amplitude. The rate of myocardial glycogenolysis is significantly correlated with the rate of increase in T-wave amplitude, quantified as T/QRS ratio. The presence of biphasic ST-segments is another indicator of foetal distress. They occur in two situations: 1) in the presence of acute hypoxic stress, when the foetal heart had no time to respond to hypoxia and 2) when the foetal heart is not capable to respond to hypoxia due to the lack of resources in (chronic) stress situations. The resulting ischaemia of the endocardium leads to altering of the repolarisation, resulting in a depression in the – normally iso-electric – ST-segment. Biphasic ST-events are graded from 1 to 3 by the degree of the depression (Amer-Wahlin and Kwee, 2016; Garabedian et al., 2017).

STAN ® monitors the foetal ECG continuously in an automated way. Changes in the ST-interval are shown on the CTG-monitoring device as “ST- events”. These events should always be evaluated in the context of the CTG-pattern: an ST-event in presence of a normal and reactive CTG should not lead to action (except continuous monitoring) as it is a normal reaction of some foetuses to the stress and effort of labour, whereas ST-events in the presence of an intermediary or abnormal CTG will require intervention. To facilitate the interpretation of ST- events, STAN ® clinical guidelines were developed (Becker et al., 2011).

The introduction of STAN ® was preceded by thoroughly fundamental and clinical research. Since its introduction, STAN ® has been under debate due to contradictory results from randomised controlled trials (RCT) regarding its effect on the rate of metabolic acidosis and operative delivery rate. Since 1993, 7 RCTs, 10 meta-analyses and more than 20 observational studies on the STAN ® -method were published. The first two large RCTs showed a trend towards a decrease in foetal metabolic acidosis (only being significant in the first one) and a significant decrease in the rate of operative delivery for foetal distress when using the STAN ® -method compared to the use of CTG alone (Westgate et al. 1993; Amer-Wåhlin et al. 2001). Three other RCTs could not detect a significant effect of STAN ® on the incidence of foetal metabolic acidosis nor the rate of vaginal operative deliveries and caesarean sections. The only conclusion that could be drawn was that the introduction of STAN ® resulted in a decrease in the rate of FBS during labour (Table I) (Ojala et al., 2006; Vayssière et al., 2007; Westerhuis et al., 2010).

**Table I:** — ­ Overview of randomised clinical trials comparing CTG only with CTG+STAN ® . CTG: cardiotocography, FBS: foetal blood sampling, NRFS: non reassuring foetal status, UA: umbilical artery.

Studies	Study population	FBS rate (CTG only vs. CTG+STAN ® )	Primary outcome	Main results
Westgate et al., 1993	N = 2434 High risk labours	9.4 vs 7.6%	Total operative delivery rate, foetal distress & metabolic acidosis (umbilical artery pH < 7.05 and BD-ecf > 12 mmol/L)	- Metabolic acidosis: trend towards lower - Total operative delivery for foetal distress: decrease - Rate of FBS: no difference
Amer-Wahlinet al., 2001	N = 4966 High risk pregnancies	11 vs 9%	Metabolic acidosis (umbilical artery pH < 7.05 and BD-ecf > 12 mmol/L)	- Metabolic acidosis: decrease - Operative delivery for foetal distress: decrease - Rate of FBS: no difference
Ojala et al., 2006	N = 1483 After amniotomy	15.6 vs 7%	Umbilicalartery pH <7.10	- Metabolic acidosis: no difference - Operative delivery rate: no difference - Rate of FBS: decrease
Vayssière et al., 2007	N = 799 Abnormal CTG or thick meconium stained amniotic fluid	62 vs 27%	Operative deliveries for foetal distress	- Operative deliveries: no difference - Rate of FBS: decrease
Westerhuis et al., 2010	N = 5681 High risk pregnancies	20.4 vs 10.6%	Metabolic acidosis (umbilical artery pH < 7.05 and BD-ecf> 12 mmol/L)	- Metabolic acidosis: no difference - Operative delivery rate: no difference - Low Apgar, neonatal encephalopathy: no difference - Rate of FBS: 48% decrease
Belfort et al., 2016	N = 11 108 >36 wks, attempting vaginal delivery, 2-7cm dilation	Not applicable	Composite outcome: - Intrapartum foetal death - Neonatal death - Apgar at 5 min ≤ 3, - Umbilical artery pH < 7.05 with BD-ecf> 12 mmol/ - Intubation or ventilation at delivery - Neonatal encephalopathy	- Primary composite outcome: no difference - Operative delivery rate: no difference
Puertas et al., 2019	N = 237 Post-term	Not applicable	Umbilical artery blood pH in neonates after abdominal or vaginal operative delivery for NRFS	- Neonatal UA pH: no difference - Caesarean delivery rate & operative delivery for NRFS: no difference

One of the most recent RCTs on STAN ® was conducted in the United States (Belfort et al., 2015). In a cohort of women with low-risk pregnancies, no difference was seen in the rate of metabolic acidosis, caesarean section or vaginal operative delivery when monitoring with CTG alone was compared to the STAN® method. A FBS was not optional in both study arms of this trial.

To date, 3 meta-analyses were published including the first 6 aforementioned RCTs. Experts have agreed that the meta-analysis by Blix et al. (2016) is the most appropriate as it is the only one relying on the correct (revised) data and using the best methodology (Amer-Wåhlin et al., 2019). This meta-analysis however concluded that STAN ® is beneficial over CTG monitoring alone, as the pooled data showed a reduction in the rate of metabolic acidosis by 36% and of vaginal operative deliveries by 8%. Based on these results, it is now stated that STAN ® is superior to CTG monitoring alone in detecting true metabolic acidosis and avoiding unnecessary vaginal operative deliveries.

## Methods

A literature search was performed in July 2019. The following scientific databases were used: Pubmed, Embase and Google Scholar. Search terms for the intervention were: fetal blood sampling, fetal scalp blood sampling, fetal scalp pH and fetal scalp lactate. Search terms for the comparison were: ST analysis, ST segment analysis, STAN, fetal electrocardiography, and for the outcome: metabolic acidosis. Search results were further refined using the terms “labour” or “pregnancy”. Clinical randomised trials and observational studies were selected and included if they met one the following criteria: randomised studies comparing the intervention CTG+STAN ® +FBS compared to CTG+STAN ® , CTG+FBS or CTG alone, observational studies where FBS was performed additionally to STAN ® and the indications, the results and outcomes (neonatal and/or maternal) were reported, follow-up of the neonates should at least have consisted of sampling of arterial and venous umbilical cord blood for blood gas analysis. Only articles written in English and published in peer reviewed journals were included. Study selection, data extraction and critical assessment of the included studies were done by a single reviewer (first author), therefore a “restricted systematic review” was performed. In “restricted systematic reviews”, certain elements required in systematic reviews are simplified or omitted. When conducted correctly, information obtained from “restricted systematic reviews” can be used for decision making regarding healthcare interventions (Plüddemann et al., 2018). This study met the minimum requirements for completing a restricted systematic review.

**Figure 1:**
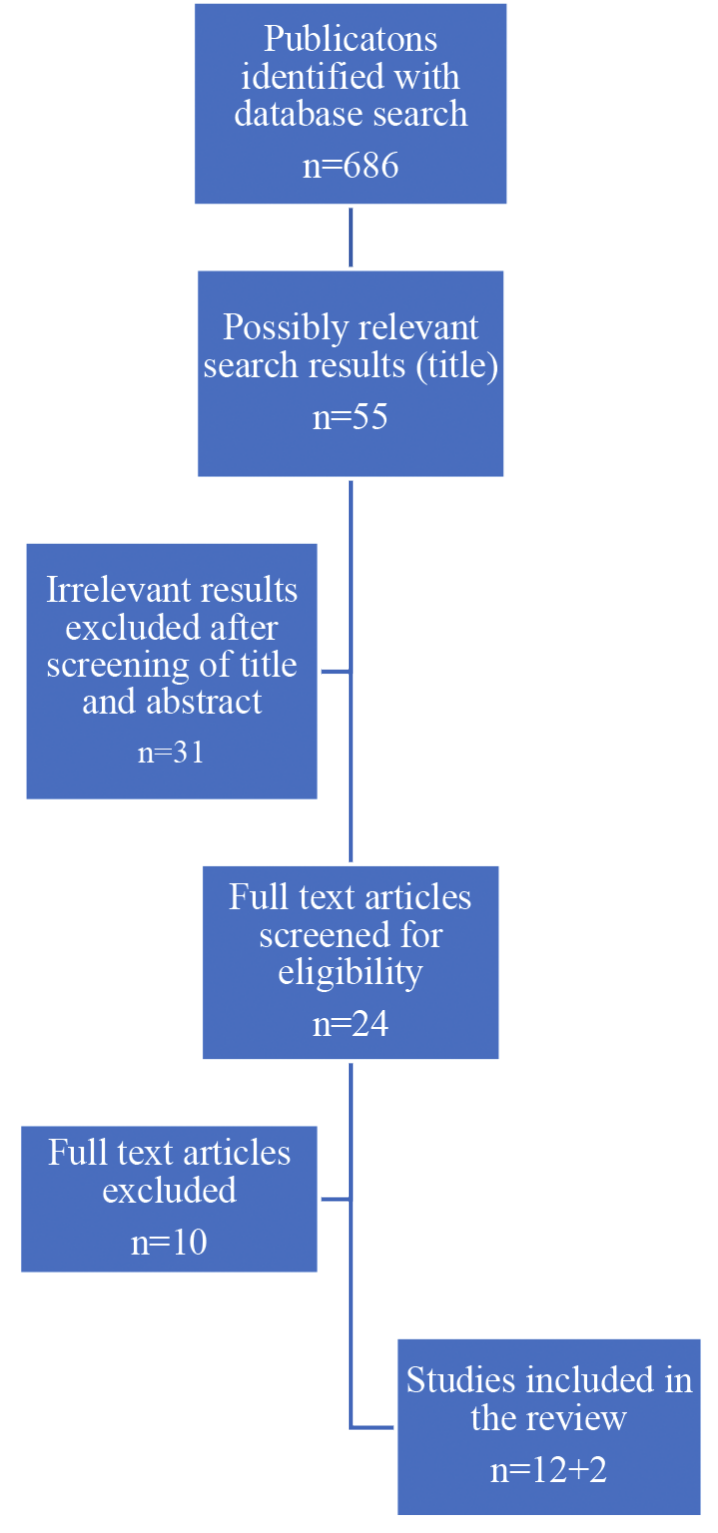
Flowchart search results and study selection. After screening of title and abstract, irrelevant results were excluded because of not meeting the inclusion criteria. Full text articles excluded after reading consisted of observational studies comparing CTG+STAN ® with CTG alone, without performing FBS, or data regarding FBS results were not provided.

Of all titles screened, 55 publications were selected for further reading of the abstract. Based on this, 24 publications were selected for evaluation of the full text. References of these articles were also assessed, and possibly relevant articles were manually searched for. Five randomised clinical trials and seven observational trials were withheld (Figure 1). To have a complete overview of all randomised clinical trials concerning STAN ® monitoring, two trials in which FBS was not performed, were also included.

## Results

To date, there are no studies directly comparing CTG+FBS with CTG+STAN ® . In five of the seven RCTs comparing CTG only with CTG+STAN ® the performance of FBS was allowed in both study groups according to clinical guidelines or clinicians’ judgement (Westgate et al., 1993; Amer-Wåhlin et al., 2001; Ojala et al., 2006; Vayssière et al., 2007; Westerhuis et al., 2010; Belfort et al., 2015; Puertas et al., 2019) (Table I). FBS rates varied from 9.4% to as much as 62% (Westgate et al., 1993; Vayssière et al., 2007). The majority of these trials showed a reduction in FBS rate when STAN ® monitoring was applied (Ojala et al., 2006; Vayssière et al., 2007; Westerhuis et al., 2010).

The Dutch RCT is the only study showing some benefit of performing a FBS in the setting of STAN ® monitoring (Westerhuis et al., 2010). In this trial, the performance of FBS was restricted to three situations: 1) start of STAN ® registration with an intermediary or abnormal CTG trace, 2) abnormal CTG trace > 60 minutes during the first stage of labour without ST events, 3) poor ECG signal quality in the presence of an intermediary or abnormal CTG trace. In these cases, where according to the STAN ® -method intervention was not needed, FBS led to the detection of foetal acidosis (defined as a scalp pH < 7.20) in 9.9% (Table II and III) (Becker et al., 2011). Neonatal metabolic acidosis was present in only three cases (1.75%) where FBS was performed according to the protocol. Two of them were performed because of an abnormal CTG > 60 minutes. In these cases however, FBS did not appear to perform well, as a normal scalp pH was found indicating that no intervention was needed. The third FBS was performed because of poor ECG signal quality in the presence of an abnormal CTG for 36 minutes. Here, foetal acidosis was correctly identified. When FBS was not performed according the protocol, a significant ST-event preceding the performance of a FBS was most predictive of foetal or neonatal acidosis.

**Table II:** — ­ Overview of observational studies concerning STAN ® with additional analysis of FBS performed.

Study	Study period	Centre	Labours monitored with STAN ®	Labours in which FBS was done (%), samples available for analysis
Kwee et al., 2004	08/2000-11/2002 monocentric	Tertiary referral centre	637 (449 analysed)	142 (22%), 192
Luttkus et al. 2004	10/2000 – 06/2002 multicentric	10 university hospitals	6999	911 (13%)
Norén et al. 2007	10/2000 – 06/2002 multicentric	10 university hospitals	6999	911 (13%)
Norén and Carlsson 2010	01/2001-12/2007 Detailed analysis of data from 2001 and 2005-2007 monocentric	Peripheral centre	7663	444 (5.8%)
Becker et al. 2011	01/2006-07/2008 multicentric	3 academic, 6 non-academic hospitals	2827	297 (10.5%) - 171 according to protocol - 126 not according to protocol
Ragupathy et al. 2010	10/2007-03/2008 monocentric	University hospital	253	39 (15%)
Kessler et al. 2013	01/2004-12/2008 monocentric	University hospital	6010	146 (2.4%), 185

**Table III:** — Overview per observational study of FBS rate, FBS results, number of labours an ST event was present in case of an abnormal FBS result and the occurrence of metabolic acidosis. FBS: foetal blood sampling, MA: metabolic acidosis: umbilical cord artery pH <7.05 and BD-efc>12 mmol/L.

Study	FBS/number of labours	FBS result			ST-events when FBS pH <7.20 was found		Cases of metabolic acidosis when abnormal FBS was found
Kwee et al., 2004	192/637	pH <7.15	10		8		No records
		pH 7.15-7.19	11		6		
		pH 7.20-7.24	30		9		
		pH ≥7.25	141		15		
Norèn and Carlsson, 2010	444/12.832 (labours in 2001 and 2005-2007)	pH<7.20	84		39		1/84 (FBS preceded by ST-event) 2/16 respiratory acidosis (instable FHR at onset STAN ® )
Becker et al., 2011	297/2827 171 according to protocol* - 126 not according to protocol**	pH<7.20	17*	10**	0*	8**	0
		pH 7.20-7.25	33*	15**	0*	5**	
		pH >7.25	112*	96**	0*	19**	
Ragupathy et al., 2010	36/253	pH<7.20	2		1		0
		pH 7.20-7.25	4		3		
		pH > 7.25	30		/		
Kessler et al., 2013	185/6010	pH<7.20	21		8		No records

Similarly, variable results were found in the seven observational studies that met the inclusion criteria (Table II, Table III) (Kwee et al., 2004; Luttkus et al., 2004; Norén et al., 2007; Norén and Carlsson, 2010; Ragupathyet al., 2010; Becker et al., 2011; Kessler et al., 2013). Two trials assessed the relationship between scalp pH and ST analysis (Luttkus et al., 2004; Norén et al., 2007). Four trials studied the effect of the clinical implementation of STAN ® monitoring and provided data regarding FBS performed (Kwee et al., 2004; Norén and Carlsson, 2010; Ragupathyet al. 2010; Kessler et al. 2013). As in the RCTs, the decision on whether to perform a FBS was left at the discretion of the physician. FBS rates ranged from 2.4 to 22% (Table II) and foetal acidosis (pH below 7.20) was found in 5.5 to 18.9% (Table III). However, FBS was often performed shortly before or even after STAN ® indicated the need for intervention and thus had no additional value. In the absence of STAN ® -events, FBS showed a non-reassuring result in 2.7 to 10.1% of the cases. Performing FBS after the occurrence of a significant ST-event is not recommended since this act will only induce more time lag between the ST-event and an intervention ( Kwee et al., 2004; Luttkus et al., 2004; Norén et al., 2007; Norén and Carlsson, 2010; Becker et al., 2011).

Data from Norén and Carlsson (2010) showed that when FBS was performed in the absence of ST- events and the result was non-reassuring, no cases of neonatal metabolic acidosis nor Apgar scores at 5 minutes of < 7 were identified. However, a non- reassuring FBS seemed to correlate well with an umbilical cord artery pH ≤ 7.15 when performed in selected situations: 1) start of STAN ® recording in second stage of labour, 2) presence of an unstable FHR at onset, 3) absence of ST data in the presence of an intermediary or abnormal CTG according to STAN ® clinical guidelines. These situations are similar to those mentioned in the Dutch RCT.

In the observational study by Kessler et al. (2013) FBS was performed if an abnormal CTG was present at the onset of STAN ® monitoring or if the physician was uncertain about the foetal wellbeing. In 7% a pH value of <7.20 was found in absence of ST-events. In four (out of 13 cases), FBS was performed because of severely impaired ECG signal quality combined with an abnormal CTG, in eight there was an abnormal CTG at onset of the STAN recording, leaving only one case rather unexplained as to why STAN ® did not indicate intervention in these cases (Kessler et al., 2013).

In contrast to the other observational studies, Norén et al. (2007) concluded that STAN ® is able to correctly identify metabolic acidosis even when registration is started in absence of a normal CTG pattern, as long as STAN ® registration is started during the first stage of labour, and continued up until 20 minutes before delivery. This was based on the finding that in 14 out of 17 cases where STAN ® registration was started in absence of a normal CTG and marked neonatal acidosis (umbilical cord artery pH <7.06) was afterwards found, STAN ® had recommended intervention prior to an abnormal FBS result.

**Table IV:** — Overview per observational study of the number of cases of metabolic acidosis, occurrence of significant ST-events in case of metabolic acidosis, number of cases in which FBS was done with abnormal result. FBS: foetal blood sampling. Abnormal FBS result: pH<7.20. Metabolic acidosis: umbilical cord artery pH<7.05 and BD-efc>12mmol/L.

Study	Number of cases with metabolic acidosis	STAN ® guidelines indicating intervention	Cases of metabolic acidosis in which FBS was done	Cases of metabolic acidosis with FBS result pH<7.20
Kwee et al., 2004	22/637 (labours monitored with STAN ® )	11	12	6
Luttkus et al., 2004	20/911 (labours monitored with STAN ® and FBS)	18	20	12
Norén et al., 2007	Idem			
Norén and Carlsson, 2010	20/10 415 (all labours in 2005-2007 irrespective of foetal monitoring technique)	11	No records	1
Becker et al., 2011	20/2827 (labours monitored with STAN ® = index group “Dutch trial”)	No records	No records	No records
	6/224 (labours available for analysis by Becker et al.,STAN ® and FBS during labour)	3	6	1
Ragupathy et al., 2010	4/253 (laboursmonitoredwithSTAN ® )	4	0	/
Kessler et al., 2013	37/6010 (laboursmonitoredwithSTAN ® )	30	No record	No records

## Discussion

The aim of this restricted systematic review was to investigate if FBS is useful in detecting and preventing metabolic acidosis when ST-analysis of the foetal ECG is already being used as a second line technique for intrapartum foetal monitoring. Our results show that only limited data are available to answer this research question.

Only analysis of data coming from one RCT showed some benefit of performing a FBS in the setting of STAN ® monitoring when performed in specific situations (Westerhuis et al., 2010; Becker et al., 2011). In the presence of one of the following: 1) start of STAN ® registration with an abnormal CTG trace, 2) abnormal CTG trace > 60 minutes during the first stage of labour without ST events, 3) poor ECG signal quality in the presence of an intermediary or abnormal CTG trace, FBS identified foetal acidosis in 9.9% of the cases where STAN ® monitoring did not indicate intervention. The real benefit of FBS is, however, limited, as only one case of true (neonatal) metabolic acidosis was correctly identified. In the majority of the other RCTs it could only be concluded that the introduction of STAN ® monitoring resulted in lower level performance of FBS.

Analogously, observational data showed that FBS is able to identify foetal acidosis in up to 10% of the cases where STAN ® monitoring is reassuring but other factors are warranting FBS. When FBS is used only in selected cases similar to those mentioned in the RCT and FBS is non-reassuring, umbilical artery pH is found to be in the lower range. The incidence of these specific situations in which an FBS could be valuable is low (3.6 to 6%) (Norén and Carlsson, 2010; Becker et al., 2011). This questions the relevance of this 10% foetal acidosis rate, especially since in all other cases STAN ® was able to correctly indicate intervention.

STAN ® clinical guidelines (2007) recommend qualified assessment of the CTG and checking for non-deteriorating foetal state if an abnormal CTG pattern is present for more than 60 minutes or less if the foetal heart rate deteriorates rapidly with normal ST analysis (Becker et al., 2011). How to check for foetal state is not defined, but FBS might be an option. Based on these results however, additionally performing FBS is uncertain in improving neonatal outcome.

Besides the fact that our results show only limited value for performing FBS, there are other reasons for questioning its use. Upon its introduction FBS has only been investigated as an adjunct to intermittent auscultation and not validated for use as an additional test to continuous CTG for assessing foetal wellbeing (Adamsons et al., 1970). Although the negative predictive value (99-100%) of FBS for foetal acidosis at birth seems high, the positive predictive value is rather low (1-12%) (Saling, 1966; Beard, 1968). Comparable to continuous CTG, the technique mainly serves to reassure the obstetrician when a normal pH or lactate level is found in the presence of a non-reassuring foetal heart rate pattern. Moreover, FBS is a discontinuous – frequently requiring multiple repetitions of the method – and invasive technique sometimes resulting in serious adverse events. Finally, as it takes several minutes to perform the technique, precious time can be lost when the foetus is truly hypoxic.

Trials regarding the STAN ® method, however, also have their limitations. In the majority of the RCTs (and observational studies) investigating STAN ® monitoring, FBS was liberally performed in both study groups. As to what extent FBS was a determining factor for the clinical decision and the foetal outcome in these trials is unknown. It is questionable if we can extrapolate these results to a setting were FBS is often not an option, like in most Flemish delivery wards. However, one trial describing the rate of operative delivery performed due to an abnormal pH on FBS, showed this rate to be the same in the two study groups (CTG versus CTG+ST-analysis) (Westgate et al., 1993). The two trials where FBS was not an option could not show any significant differences in neonatal and maternal outcomes, suggesting FBS did not influence results (Belfort et al., 2015; Puertas et al., 2019).

Another form of bias is the Hawthorne effect, the tendency for people to perform better when they believe they are being watched, when for example participating in a clinical trial. In addition, most trials were conducted shortly after the STAN® method was implemented in obstetric practice, which was associated with intense training in CTG interpretation and ST-analysis (classifying the CTG pattern as normal, intermediary or abnormal is decisive for the interpretation of ST-events). This might be the explanation for the often lower than anticipated incidence of the primary outcome in the control group in these trials, since results were also better in the control group (CTG only) due to improved CTG interpretation skills. This left these trials often underpowered regarding these outcomes. Both phenomenons were illustrated in an observational study by Landman et al. (2019) as well. Over a 14-year period, an 84% reduction in the incidence of umbilical artery acidosis was shown after STAN ® was introduced, with the greatest share of the decrease taking place when this obstetric centre was participating in the Dutch trial (Westerhuis et al., 2010). During the Dutch trial itself however, no significant differences were shown in neonatal outcomes when STAN ® was compared with CTG only. Moreover, with increasing use of STAN ® (from 20% of deliveries during the Dutch trial to nearly all deliveries by 2010) metabolic acidosis rate did not decrease further, implying that other factors besides STAN ® monitoring were responsible for these improved neonatal outcomes.

This review has some limitations. Although results coming from restricted systematic reviews were shown not to differ too much from results from systematic reviews, they will probably result in more cautious interpretation and recommendations (Plüddemann et al., 2018). Only one study included had the same research question (Becker et al., 2011). Other studies were not designed to answer this research question. Moreover, results were presented in different ways (e.g. analysis of only non-reassuring FBS results versus analysis of results of FBS in cases where metabolic acidosis was found afterwards), making comparison between results difficult.

## Conclusion

This restricted systematic review was performed to study the effect of FBS when STAN ® monitoring is already being used as second line technique for intrapartum foetal monitoring. Although FBS was able to identify foetal acidosis in up to 10% of the cases where ST-events were absent, a non- reassuring FBS was not at all predictive of neonatal metabolic acidosis. Based on current evidence no recommendations in favour of combining FBS with foetal monitoring consisting of CTG and STAN ® monitoring can be made.
